# Implementation science: Epidemiology and feeding profiles of the Chagas vector *Triatoma dimidiata* prior to Ecohealth intervention for three locations in Central America

**DOI:** 10.1371/journal.pntd.0006952

**Published:** 2018-11-28

**Authors:** Raquel Asunción Lima-Cordón, Lori Stevens, Elizabeth Solórzano Ortíz, Gabriela Anaité Rodas, Salvador Castellanos, Antonieta Rodas, Vianney Abrego, Concepción Zúniga Valeriano, María Carlota Monroy

**Affiliations:** 1 The Applied Entomology and Parasitology Laboratory at Biology School, Pharmacy Faculty, San Carlos University of Guatemala, Guatemala City, Guatemala; 2 Department of Biology, University of Vermont, Burlington, Vermont, United States of America; 3 Centro de Investigación y desarrollo en salud (CENSALUD-CID), Universidad de El Salvador, San Salvador, El Salvador; 4 Departamento de Vigilancia de la Salud, Hospital Escuela Universitario, Tegucigalpa, Honduras; Universidad del Valle de Guatemala, GUATEMALA

## Abstract

The Ecohealth strategy is a multidisciplinary data-driven approach used to improve the quality of people’s lives in Chagas disease endemic areas, such as regions of Central America. Chagas is a vector-borne disease caused by the parasite *Trypanosoma cruzi*. In Central America, the main vector is *Triatoma dimidiata*. Because successful implementation of the Ecohealth approach reduced home infestation in Jutiapa department, Guatemala, it was scaled-up to three localities, one in each of three Central American countries (Texistepeque, El Salvador; San Marcos de la Sierra, Honduras and Olopa, Guatemala). As a basis for the house improvement phase of the Ecohealth program, we determined if the localities differ in the role of sylvatic, synanthropic and domestic animals in the Chagas transmission cycle by measuring entomological indices, blood meal sources and parasite infection from vectors collected in and around houses. The Polymerase Chain Reaction (PCR) with taxa specific primers to detect both, blood sources and parasite infection, was used to assess 71 *T*. *dimidiata* from Texistepeque, 84 from San Marcos de la Sierra and 568 from Olopa. Our results show that infestation (12.98%) and colonization (8.95%) indices were highest in Olopa; whereas *T*. *cruzi* prevalence was higher in Texistepeque and San Marcos de la Sierra (>40%) than Olopa (8%). The blood meal source profiles showed that in Olopa, opossum might be important in linking the sylvatic and domestic Chagas transmission cycle, whereas in San Marcos de la Sierra dogs play a major role in maintaining domestic transmission. For Texistepeque, bird was the major blood meal source followed by human. When examining the different life stages, we found that in Olopa, the proportion bugs infected with *T*. *cruzi* is higher in adults than nymphs. These findings highlight the importance of location-based recommendations for decreasing human-vector contact in the control of Chagas disease.

## Author summary

Blood feeding insects from the subfamily Triatomine are involved in the transmission of Chagas disease, caused by the protozoan parasite *Trypanosoma cruzi*, a neglected tropical disease endemic from southern Mexico through Central to northern South America. Chagas disease mostly affects rural areas and especially people living in houses made of low-cost, natural materials such as bajareque or adobe that have mud walls and a dirt floor. A multidisciplinary data-driven Ecohealth vector control program that includes house improvements (wall plastering and cement flooring), as well as insecticide spraying, was developed in Jutiapa department, Guatemala, and has been shown to decrease vector-human contact. Because Chagas vectors feed on a wide variety of vertebrates, knowing the local feeding profiles of the insect vectors before interventions can strengthen Ecohealth program development. To facilitate scaling up the Ecohealth program developed in Jutiapa to three new locations in three different countries, Texistepeque, El Salvador; San Marcos de la Sierra, Honduras and Olopa, Guatemala, and with distinct ecological scenarios, we assessed the entomological indices, feeding profiles and parasite infection of vectors collected in and around houses in the new locations prior to any interventions. Our results show all three metrics varied among locations. The results highlight the importance of domestic, synanthropic and sylvatic blood meal sources on the disease transmission cycle and the need to consider local conditions for vector control.

## Introduction

Chagas disease or American trypanosomiasis, is a neglected, zoonotic vector-borne disease, transmitted by insect vectors in the subfamily Triatominae (Hemiptera: Reduviidae) known colloquially as “kissing bugs” [1, 2]. The disease, caused by the parasite *Trypanosoma cruzi* (Kinetoplastea: Trypanosomatida), is endemic throughout Latin America with some autochthonous cases reported to the southern United States [3–6]. It has been estimated that in 2010, 5.7 million people from 21 Latin America countries were infected with *T*. *cruzi* [1, 2]. Among the different pathways to acquire the disease (e.g., mother-fetus, oral, blood transfusion, organ transplant), insect vector transmission is the most common, and there are over 150 species of triatomine vectors distributed across the endemic area [7]. For Central America, *Triatoma dimidiata* became the most important vector in the human Chagas transmission cycle after vector control strategies successfully eliminated *Rhodnius prolixus*, a vector indigenous to South America that had been introduced into Central America [8–10]. In its introduced range, *R*. *prolixus* was exclusively domestic, whereas *T*. *dimidiata*, a vector native from southern Mexico through Central America and into northern South America, is a complex of subspecies found in sylvatic and domestic habitats [11–16].

International health organizations (i.e. Pan American Health Organization, PAHO and World Heath Organization, WHO) identify reducing vector infestation as the main way to reduce human Chagas disease[17]. To this end, a multifactorial Ecohealth strategy combining insecticide spraying and house improvements has been developed that eliminates vector hiding places within and around houses to decrease the presence of bugs, reduce vector-human contact, and improve the quality of people’s lives [18–20]. The biology and ethology of *T*. *dimidiata* have been well studied [20–25] and its domiciliary infestation has been associated with different bio-socio-ecological factors. These bio-socio-ecological factors include: I) the presence of domestic and synanthropic animals, such as bird (e.g., chicken, turkey and duck), rodents (mouse and rat), dog, and opossum, [20, 22–24, 26]; II) house construction with natural materials specifically adobe or bajareque, and house wall conditions including rustic, unplastered walls or cracks in the wall plastering [27]; III) the location of chicken coops (next to the house or away from the house) or evidence of animals inside the house (i.e. rodent or bird nests), and IV) household characteristics including: the presence of dirt floors, poor hygiene (e.g., clutter), and signs of triatomines inside the house (insect feces, exuviae, eggs or dead insects) [26–30]. House improvements that target these factors include not only replacing dirt floors with concrete and plastering the walls using local materials, but also removing blood meal sources by removing clutter and relocating chicken coops and other domestic animals outside and away from the house.

An essential component of the Ecohealth approach is the collection of data before, during, and after intervention (data-driven intervention), to enable data-driven evaluation. This research-based approach was developed and tested in Jutiapa, Guatemala [24]. Before and after the house improvements, two common entomological indices were used to assess vector abundance: infestation and colonization indices [18]. Entomological surveys have shown these indices are reduced by the interventions of the Ecohealth strategy [26].

To identify risk factors and assessing the success of the Ecohealth interventions in a single village in Jutiapa, seven potential blood meal sources of *T*. *dimidiata* before and after house improvements were compared [20]. Within the framework of the Implementation Science approach [31], recent efforts have scaled up the Ecohealth program from the single locality in Jutiapa to three new locations in three different countries, Texistepeque, El Salvador; San Marcos de la Sierra, Honduras and Olopa, Guatemala. These localities differ in ecology, culture, ethnicities and social administrative structure.

The first step of Ecohealth interventions is assessing the initial conditions at the locations targeted for vector control. Establishing the baseline conditions of an ecosystemic intervention enhances vector control success by identifying risk factors for house infestation, and has been well reviewed [31]. The risk factors associated with domestic infestation for these localities have been identified [27]. Based on these risk factors, the aim of the present study was to: 1) document and understand the role of domestic, synanthropic and sylvatic animals in the occurrence of Chagas vector infestation in each location, and 2) use a research-based approach to determine if the locations vary in *T*. *cruzi* transmission risk factors and infestation indices prior to the Ecohealth interventions. With these goals, we surveyed the totality of houses at the three locations and collected all vectors found during half an hour, to estimate the infestation and colonization indices. From these vectors, we identified the blood source profiles based on the seven blood meal sources previously studied [20] and assessed *T*. *cruzi* infection prevalence in vectors for each of the three locations. Our data analysis tested for differences among locations evaluated in the metrics of vector infestation and infection, including vector developmental stage and ecotope of collection, as well as the association between vectors infected with *T*. *cruzi* and the various blood sources detected.

## Methods and materials

### Study locations

**Fig 1:**
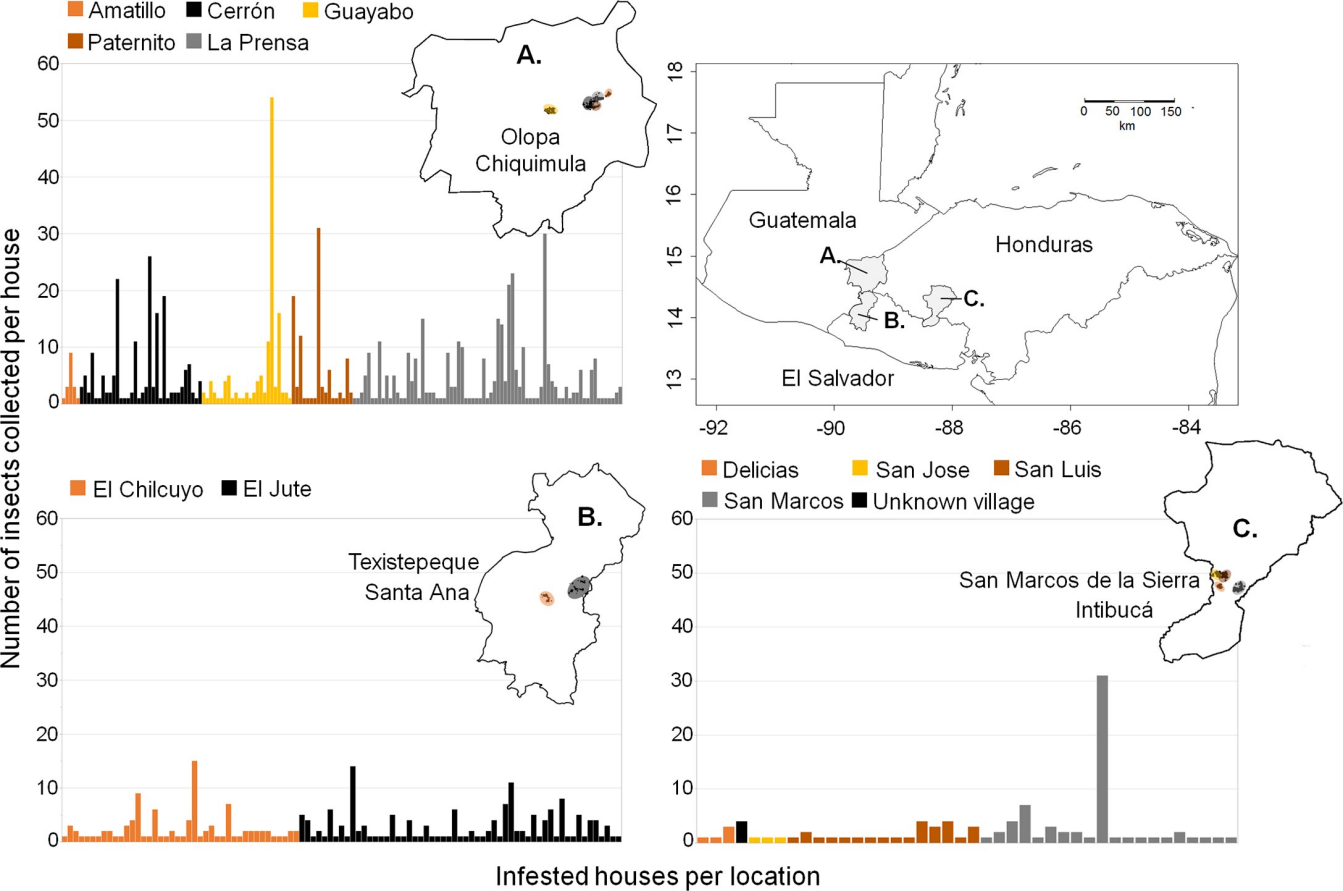
Geographical locations and histograms showing the number of bugs collected per infested houses at the three municipalities, departments and countries. Inserted maps represent each location (**A:** Olopa, Chiquimula; **B:** Texistepeque, Santa Ana and **C:** San Marcos de la Sierra, Intibucá) and colored dots represent houses within each village or canton. Histograms show the number of insects collected per infested house and the histogram colors indicate the locations in the inserted maps. The maps were created by RL and LS, specifically for this publication using the software package R. We obtained shapefiles from the GADM database of Global Administrative areas (http://gadm.org/) downloaded July 24, 2017 and house coordinates from this study.

This study was part of the project “*Ecohealth interventions for Chagas Disease prevention in Central America*” [27]. Using the Implementation Science approach [31] to increase what is known about Chagas disease and reduce transmission, interventions that mitigate factors associated with vector infestation were scaled up from one locality in Guatemala to localities in three different countries (Texistepeque, El Salvador; San Marcos de la Sierra, Honduras and Olopa, Guatemala) in Central America (Fig 1). The localities were identified by local ministry of health officials as having high incidence of vector infestation. The environmental differences among the three locations are described below.

In the municipality of Texistepeque, Santa Ana, El Salvador, is hot and dry with average annual temperature of 24.4°C and precipitation of 1,653 mm. The ecosystem is deciduous forest characterized by sandy soil with low fertility. The forest has been altered with extensive cutting and the introduction of non-native species. Most people work in agriculture (mainly growing peanuts) and small business. The houses in El Salvador are grouped in small “Caseríos” within “Cantones”, where most of the houses are constructed of adobe, block, wood or corrugated aluminum. Within Texistepeque, 928 houses from two cantons, El Jute and Chilcuyo were examined [27]. Information about prior vector control interventions (e.g. insecticide spraying) is not available for these cantons. From the 16 acute cases reported for El Salvador for 2012, seven (43.7%) came from the department of Santa Ana which includes Texistepeque [32].

The municipality of San Marcos de la Sierra, Intibucá, Honduras, is hot with average annual temperature of 21°C and average annual precipitation of 1,943 mm. The ecosystem is mountainous pine-oak forest and sandy soil with low fertility. In this area, subsistence farming is mostly corn and beans. The houses in this locality are further apart than those examined in El Salvador, most are constructed of adobe blocks with clay tile roofs. In Intibucá, we examined 613 houses from four cantones [27]. Information about prior insecticide spraying or disease prevalence is not available for this part of Honduras.

In the municipality of Olopa, Chiquimula, Guatemala, we examined five villages along a forested altitudinal gradient with the average temperature of 20°C and annual precipitation of 1,439.4 mm. The cloud forest at the highest altitude has cold weather and water year-round, while the lowest village is in humid tropical forest. Along the gradient, crops include shade grown coffee interspersed with plantations of bananas along with remnants of the original forest. Most of the houses are constructed of adobe bricks or bajareque. In Olopa we examined 1,140 houses from the five villages El Amatillo, La Prensa, El Cerrón, El Guayabo and Paternito [27]. For the five villages, the most recent insecticide sprayings prior to this study were: El Amatillo in 2004, La Prensa in 2000, El Cerrón in 2001, El Guayabo in 2001 and El Paternito in 2004 (Personal communication, Vectores program, Ministry of Health, Chiquimula, Guatemala). Information about the number of acute cases is not available for these villages, however for 2003 a Chagas disease seroprevalence of 6.52 on school-age children was reported for the department of Chiquimula which includes Olopa [33].

Animal household practices are similar for the three locations; the most frequent domesticated animals are birds (usually chickens), followed by dogs, cats and pigs, only a few houses have beasts (e.g. cows, sheep). Prior to our Ecohealth interventions, over 75% of people at the three localities kept chickens inside the house at night to prevent theft, wandering off, or predation. Dogs are almost exclusively outside guarding the house at night. Over 50% of the houses at Texistepeque and Olopa showed signs of synanthropic animals (e.g. mice and/or rats) inside the house, while only 25% of the houses at San Marcos de la Sierra reported traces of synanthropic animals. No records are available related to the presence and abundance of sylvatic reservoirs at the three localities.

### Study design

The insect vectors examined in this study were collected during the baseline survey of all the houses in each locality conducted during August-October 2011, prior to interventions which included insecticide spraying and house improvement (for more details refer to [27]). Surveys were conducted by professionals of the Ministries of Health (El Salvador, Guatemala and Honduras), members of the Laboratory of Applied Entomology and Parasitology (Guatemala), and personnel from the Center of Health Research and Development (CENSALUD) in El Salvador. Collection of triatomines was performed using the person-hour method, with two people searching all areas of a house for half an hour each using flashlight and forceps [34]. The house areas inspected included the intradomicile and peridomicile, where the peridomicile could include structures close to the house, e.g. chicken coops, as well as piles of wood or other accumulated material.

**Fig 2:**
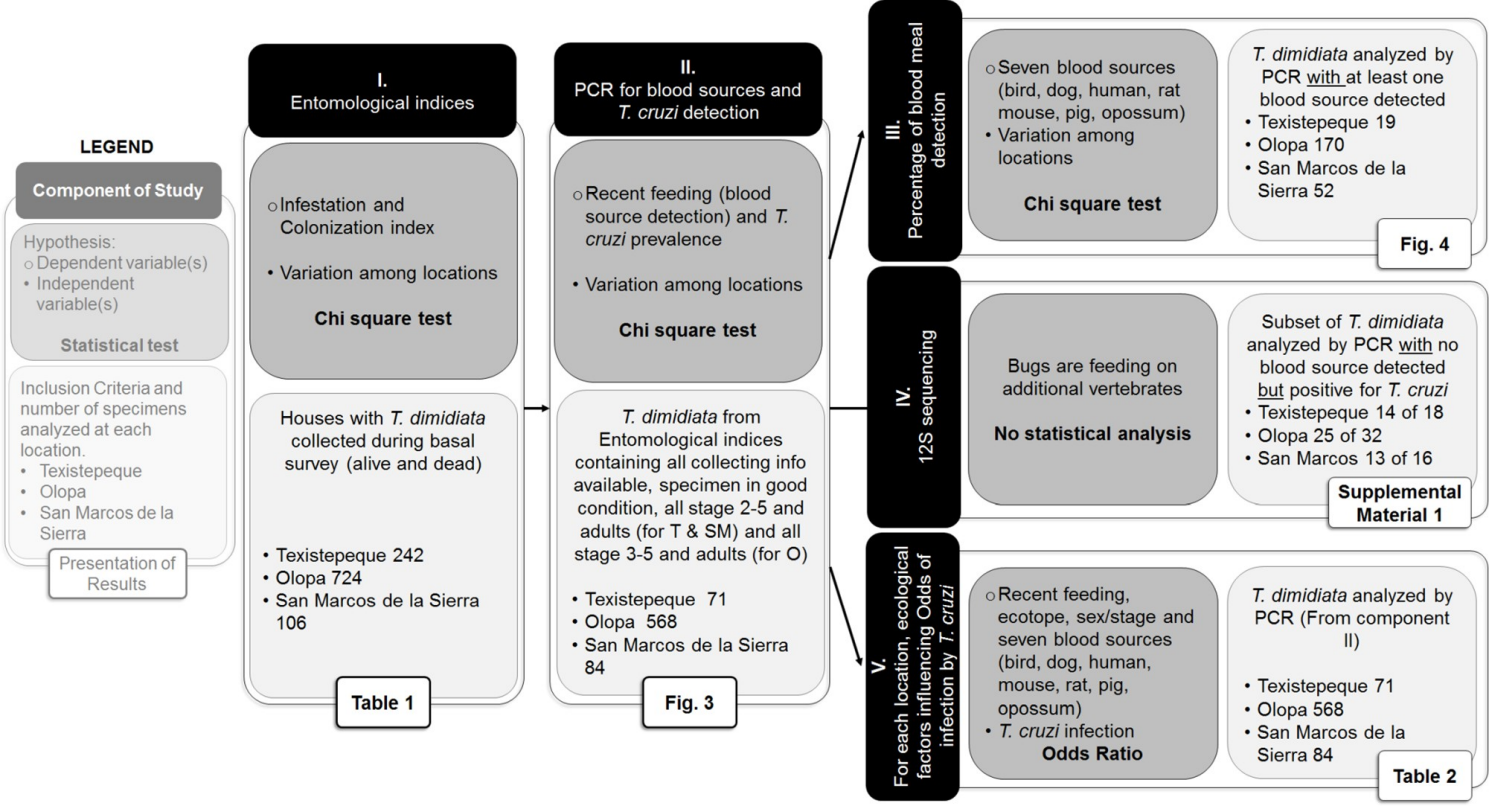
Study design. Sequential components of the study (black boxes), data analysis and statistical test for each component (dark grey box), the number of specimens included in each component (light grey box) and results associated with each component (white boxes).

All insects collected were transported to laboratories in their respective countries in mesh covered plastic bottles labeled with the collecting information (House ID, village, date, ecotope, stage). Upon arrival, collection information for each insect was recorded in an electronic database, and insect vectors were placed in 95% ethanol + 5% glycerol and stored at room temperature until subsequent DNA extraction for blood source and *T*. *cruzi* parasite detection. The sample sizes for each component of the study is shown in Fig 2.

### Parasite and blood source detection by PCR using taxa specific primers and mitochondrial 12S ribosomal sequencing

To determine the blood meal sources of insect vectors collected at houses from the three locations, we used seven PCR reactions for each individual to test for the presence of DNA from bird, dog and pig (domestic animals); rat and mouse (synanthropic animals), opossum (sylvatic reservoir) and human. For bird, we did not distinguish between domesticated (chicken, duck, turkey) and sylvatic birds. For specimens collected in Olopa and San Marcos de la Sierra, DNA was extracted in Guatemala, while for Texistepeque DNA extractions were carried in that country. All DNA extractions used the last three segments of the insect abdomen with the E.Z.N.A Tissue DNA kit (Omega Bio-Tek, Georgia, GA, USA), following the manufacturer’s tissue protocol for the first two steps, and the blood protocol for the remaining steps, with an additional incubation time at 65°C of 10 min followed by 95°C for 5 min after the third step. Positive controls for the blood meal sources were obtained from the tissue of chicken (for the bird assay) and pig, while blood was used for rat, mouse, dog, opossum, and human. All subsequent PCR assays were done in Guatemala.

Extracted DNA was used in 12 μL PCR reactions consisting of 4.5 μL H_2_O (molecular grade, DNase and RNase free), 0.5 μL of each primer (10ng/μL), 6.5 μL of 2X master mix (EconoTaq PlusGreen, Lucigen Corporation, Middleton, WI, USA, or REDTaq ReadyMix PCR Reaction Mix, Sigma, St. Louis, MO, USA) and 0.5 μL of genomic DNA (concentration not determined). Assay conditions for the *T*. *cruzi* major nuclear repetitive element followed the protocol of [35]; bird (“avian”), rat, mouse were based on [36], pig was based on [37] and human was based on the protocol of [38] (see also [20, 39]) and dog assay was based on [40]. A new assay for opossum was developed: forward primer: 5’ GATGGAGATTAGTGGCTCTG 3’, reverse primer: 5’ GAAGGCAGAGAATTCCAAGA 3’ with a PCR product size of 243 bp. PCR conditions for opossum were: denaturation at 94°C for 2 min followed by 30 cycles at 95°C (30 sec.), 50°C (30 sec) and 70°C (45 sec); followed by a final extension at 72°C for 5 minutes (S1 Text).

The PCR reactions for *T*. *cruzi*, bird, dog, human, mouse, rat, and pig were carried out using a PTC-100 thermocycler (MJ Research, California, CA, USA). For the opossum PCR reactions we used the SimpliAmp Thermal Cycler (Life Technologies Corporation, Carlsbad, CA, USA). Electrophoresis of the amplified DNA used 1% agarose gels with 10 μg/mL of ethidium bromide in TBE (90 mM Tris-borate, 1 mM EDTA, pH 8,0), followed by UV transillumination to observe the DNA bands. The opossum assays were run in 2% agarose gels stained with 2.8 μL/100 ml Syber green DNA gel stain (ThermoFisher, Waltham, MA, USA). The results (absence or presence of taxa-specific size bands) for each blood source were recorded in the electronic database.

Recent studies have shown that the lack of blood source detection by PCR can indicate either a recent blood meal from taxa not included in the survey or no recent blood meal rather than PCR inhibition [4, 20, 41]. Because of this, a subset of bugs with no blood meal detected by PCR, but positive for *T*. *cruzi*, were assayed by PCR with the universal 12S ribosomal gene vertebrate primers as in [4]. Samples with an appropriate sized band were sent for DNA sequencing in one direction (Beckman Coulter Genomics, now GeneWiz, Cambridge, MA, USA) as in [42]. Trace files were edited using Sequencher v5.3 (Gene Codes Corporation, Ann Arbor, MI USA) and taxonomically identified based on >98% match for 130 bp using NCBI-BLAST (http://blast.ncbi.nlm.nih.gov/Blast.cgi).

### Ethics statement

This study received ethical clearance for the three countries from the Panamerican Health Organization (ID: PAHO-2011-08-0017.R1). All household adult participants and parents or legal guardians of minors provided informed consent.

### Statistical analysis

The sample sizes for each component of the study is shown in Fig 2. Our sample sizes differ slightly from those reported in the study of socioeconomic and house construction factors for these same houses [27] because we included dead insects in the analysis. Although we sampled all the houses within each village, canton or caserio at each of the three localities; because of sample sizes differences among villages and caserios/cantones we pooled the data into the three localities (Fig 1). Thus for example, we cannot infer that birds were the most common blood source in every canton in Texistepeque compared to every canton in San Marcos de la Sierra, but we can conclude that on average bird blood meals are more common in Texistepeque than San Marcos de la Sierra. All statistical analyses were run using the software JMP Pro 13.0.0 (64-bit, SAS Institute Inc., Cary, NC, 1989–2017).

#### Entomological indicators

To estimate vector abundance in each location, two frequently used entomological indices were used (see [20]): Infestation Index defined as the number of houses with *T*. *dimidiata* / total number of houses surveyed * 100, and colonization index defined as the number of houses with *T*. *dimidiata* nymphs / total number of houses surveyed *100 [1]. Among locations evaluated the indices were compared with a Chi-squared (*χ*^2^) test. The *χ*^2^ values are presented with their probability (*p)* values.

#### Blood meal source profiles

The frequencies of the blood sources (dependent variables bird, dog, mouse, human, rat, pig and opossum) were compared among locations (independent variable) with a *χ*^2^ test using only the subset of bugs with at least one blood source detected. The *χ*^2^ results are presented with their *p-values*. Blood meal source profiles are shown stratified by ecotope in the supplemental material (S1 Fig). Because of the small sample size, results from the 12S sequencing are shown in a descriptive manner.

#### Odds of *T*. *cruzi* infection based on location, recent feeding, ecotope, stage/sex and blood source

We used an odds-ratio test to compare *T*. *cruzi* infection rates among localities and to examine four independent variables that may influence the likelihood of a bug being infected (dependent variable): detection of at least one blood source (recent feeding), ecotope (intradomestic or peridomestic), sex/stage (stage III-V nymph, male or female) and blood source (bird, dog, mouse, human, rat, pig and opossum) using a nominal logistic regression. We tested for a relationship between blood meal sources and *T*. *cruzi* infection by classifying the blood meal sources based on the type of Ecohealth intervention required. The grouping was done because of small sample sizes for some blood meal sources and reflected the role in the transmission cycle: I) human, II) synanthropic animals (mouse and rat), III) domestic animals (dog and pig) and IV) sylvatic (Opossum). Human blood meals were considered separately because of their inherent interest. Mouse and rat were considered synanthropic because traces of both were found inside the houses during the surveys, homeowners reported seeing them in houses and surveyors observed them in houses. Opossum was considered a sylvatic blood source because no traces were found in the houses, no homeowners reported seeing them in houses and surveyors never observed them in houses. Odds ratio (OR) results are presented with 95% confidence intervals (CI) and *p-values*. Because first stage nymphs (all three locations) and some second stage nymphs (Olopa) were excluded from the PCR assays, only stage III-V nymphs are included in the comparative analyses.

## Results

The major findings were: (1) infestation and colonization indices varied among localities with the highest values in Olopa; (2) *T*. *cruzi* prevalence and recent feeding (any blood source detected) differed among localities, with the highest parasite and blood source detection in San Marcos de la Sierra; (3) blood source profiles differ among localities, all three locations showed similar feeding on humans; however Olopa differed in having a sylvatic feeding source, opossum; while Texistepeque, El Salvador and San Marcos de la Sierra show significantly higher feeding on a synanthropic blood source, mouse; (4) 12S sequencing reveals additional blood sources and (5) *T*. *cruzi* infection is associated with different ecological and life history factors in each location. We elaborate on these findings below.

### *T*. *dimidiata* entomological indicators for the three locations

Of the 2681 houses surveyed across the three locations, Olopa had significantly higher infestation than San Marcos de la Sierra and Texistepeque (*χ*^2^ = 13.84; *p =* 0.001); Olopa also had a significantly higher colonization index than San Marcos de la Sierra and Texistepeque (*χ*^2^ = 32.76; *p* < 0.001). For all three locations, bugs were found in both the intradomestic and peridomestic ecotope, whereas dead bugs were only found inside houses (Table 1).

**Table 1:** Distribution of houses surveyed, entomological indices and total number of bugs collected and analyzed by PCR for the three localities surveyed in August-September 2011.

		Department, municipality		
Study component		Santa Ana, Texistepeque	Chiquimula, Olopa	Intibucá, San Marcos de la Sierra
**Component I**				
**Houses surveyed**		928	1140	613
**Infestation index**				
Number of houses with *T*. *dimidiata* (alive and dead) / Total of houses surveyed (%)		81/928 (8.73)	148/1140 (12.98*)	45/613 (7.34)
**Colonization index**				
Houses with *T*. *dimidiata* nymphs (alive and dead) / Total of houses surveyed (%)		29/928 (3.13)	102/1140 (8.95*)	26/613 (4.24)
**Total number of bugs collected during basal survey**		242	724	106
Alive	Intradomestic (%)	117/242 (48.34)	648/724 (89.50)	65/106 (61.32)
	Peridomestic (%)	52/242 (21.48)	60/724 (8.29)	12/106 (11.32)
	Ecotope not reported (%)	-	-	22/106 (20.75)
Dead	Intradomestic (%)	73/242 (30.17)	16/724 (2.21)	7/106 (6.60)
	Peridomestic (%)	-	-	-
	Ecotope not reported	-	-	-
**Component II**				
**Total number of bugs analyzed by PCR from Component I (%)**		71/242 (29.34)	568/724 (78.45)	84/106 (79.24)
Alive	Intradomestic analyzed by PCR / Total intradomestic collected (%)	49/117 (41.88)	503/648 (77.62)	65/65 (100.00)
	Peridomestic analyzed by PCR / Total intradomestic collected (%)	12/52 (23.07)	49/60 (81.66)	12/12 (100.00)
	Ecotope not reported analyzed by PCR / Total with ecotope not reported (%)	-	-	0/22 (0.00)
Dead	Intradomestic analyzed by PCR / Total intradomestic collected (%)	10/73 (13.69)	16/16 (100.00)	7/7 (100.00)
	Peridomestic analyzed by PCR / Total intradomestic collected (%)	-	-	-
	Ecotope not reported analyzed by PCR / Total with ecotope not reported (%)	-	-	-

*Indicates values are significantly different (p < 0.001) than the other locations.

Overall, the distribution of potential blood sources at the three locations shows little variation. There are between 4–5 people per house at each locality and on average at least 2 dogs per house. Pigs were the least frequent overall at houses, with an average number between 2–3 per house, however the average number of birds was 9–15 per house (this includes chickens). For the three locations, more than 70% of the total houses surveyed had chickens. From the houses with chickens, 82%, 56% and 35% (Texistepeque, San Marcos de la Sierra and Olopa, respectively) do not have a facility to keep them (e.g. chicken coops), instead birds were kept inside the house. In contrast, for the 70% of houses with chickens and chicken coops, the chicken coops are located away from the house (Table 2).

**Table 2:** Distribution of potential blood sources and peridomestic risk factors for the three localities surveyed in August-September 2011.

Department, municipality		Santa Ana, Texistepeque	Chiquimula, Olopa	Intibucá, San Marcos de la Sierra
**Average number of potential blood sources (standard deviation)**				
Humans		4.03 (2.22)	4.36 (2.87)	4.92 (2.77)
Dogs		1.25 (1.58)	1.57 (1.63)	1.54 (1.26)
Pigs		0.26 (1.24)	0.20 (1.11)	0.38 (0.97)
Birds (includes chickens)		11.20 (18.26)	8.75 (8.71)	8.66 (7.86)
**Number of houses with birds (includes chickens) (%)**		693/928 (74.67)	793/1140 (69.56)	433/613 (70.63)
With No chicken coop		569/693 (82.1)	285/793 (35.71)	245/433 (56.58)
With chicken coop		124/693 (17.9)	508/793 (63.73)	188/433 (43.41)
	Inside-adjacent	36/124 (29.03)	96/508 (18.75)	11/188 (5.85)
	Outside	88/124 (70.97)	412/508 (81.10)	177/188 (94.14)
**Number of houses with other peridomestic risk factors and its location (%)**				
Firewood piles		538/928 (57.97)	581/1140 (50.96)	206/613 (33.60)
	Inside-adjacent	308/538 (57.25)	570 (98.10)	134 (65.04)
	Outside	230/538 (42.75)	11 (1.90)	72 (34.96)
Construction material		270/928 (29.09)	265/1140 (23.24)	154/613 (25.12)
	Inside-adjacent	182/270 (67.40)	232/265 (87.54)	122/154 (79.22)
	Outside	88/270 (32.60)	33/265 (12.46)	32/154 (20.78)

In addition to chicken coops, two other peridomestic risk factors were frequent at the three localities, these were firewood piles and construction materials. Firewood piles were present in more than 50% of the total houses surveyed in Texistepeque and Olopa and located next to the house; whereas only 33% of the total houses surveyed in San Marcos de la Sierra had firewood piles and 65% of them were located next to the house. Construction materials were present in less than 29% of the total houses surveyed in the three locations, however more than 67% of the houses with construction materials had those materials next to the house (Table 2).

### Blood meal source profiles differ among localities

**Fig 3:**
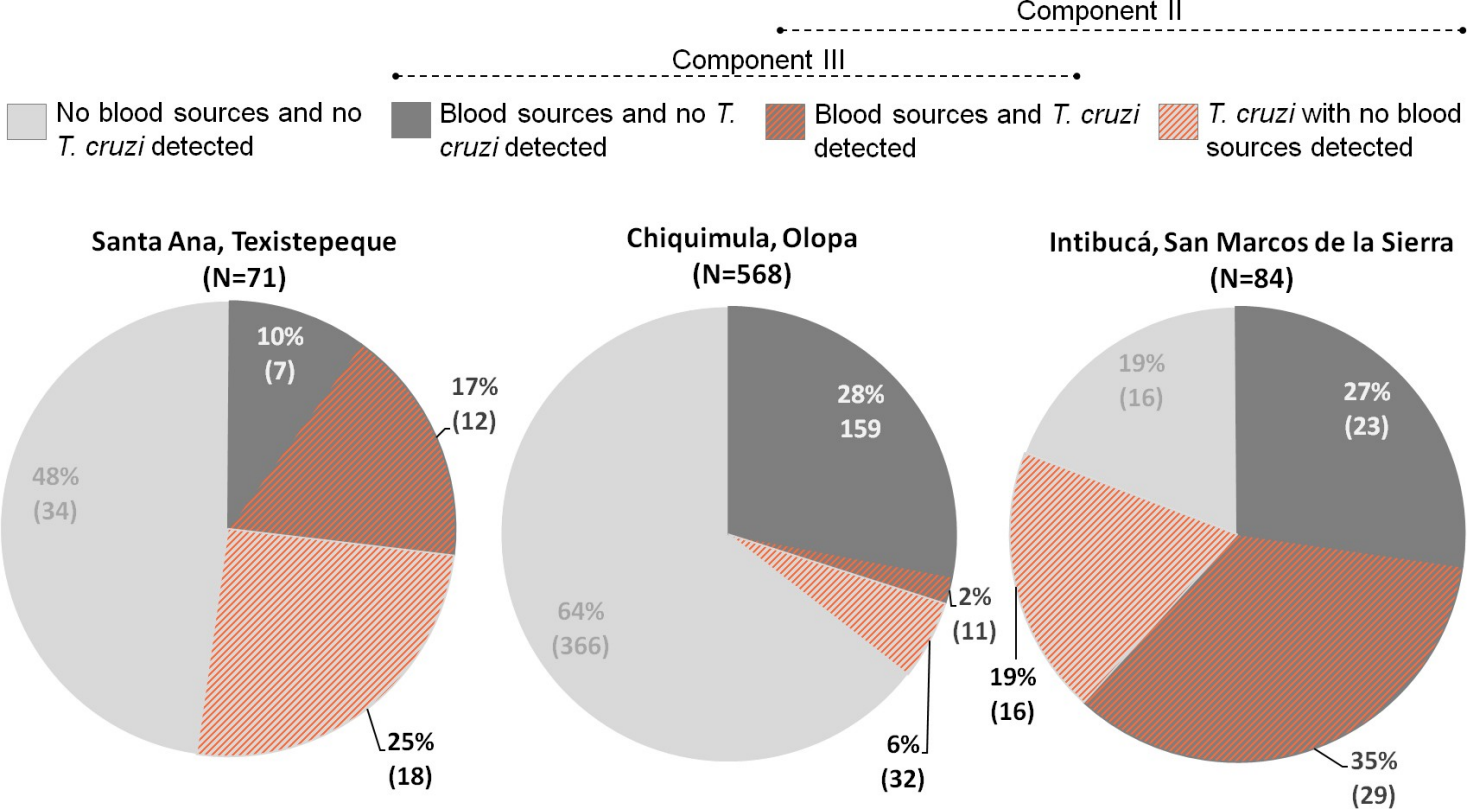
Blood sources and *Trypanosoma cruzi* infection summary for bugs analyzed from each location. Percentage of *Triatoma dimidiata* positive or negative by PCR for blood sources and *T*. *cruzi* prevalence from the three localities: Texistepeque, Olopa, and San Marcos de la Sierra. Components II and III refer to Fig 2.

The frequency of bugs from which any blood source was detected was different among localities (*χ*^2^ = 35.19; *p* < 0.001). For Texistepeque, 19 of 71 bugs (27%) had at least one blood source detected, the value for San Marcos de la Sierra was 52 of 84 (62%) and for Olopa 170 of 568 (30%) (Fig 3). Usually a single blood source was detected; however, two blood sources were detected in some bugs, [Texistepeque: 3 of 19 (16%), Olopa: 12 of 170 (7%) and San Marcos de la Sierra: 10 of 52 (19%), S1 Table column P]. More than two blood sources were not detected at any locality probably because blood meals are detected by these primers for only about 12–28 days post feeding [41].

**Fig 4:**
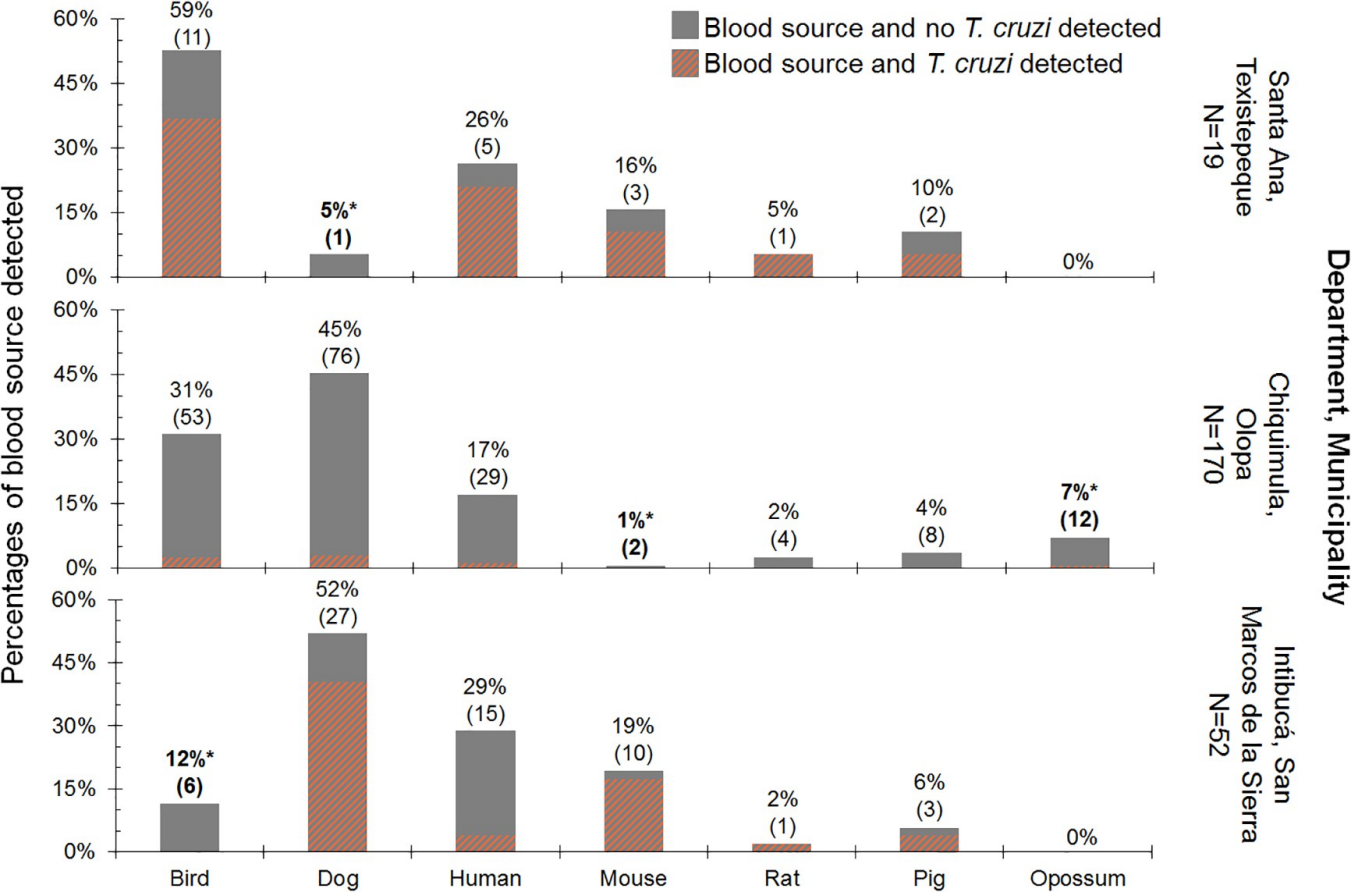
Percent of blood meals detection for each of the seven taxa assayed by PCR in *T*. *dimidiata* from the three locations (Fig 2, Component III). Note percentages sum to slightly more than 100 because of multiple blood meals detected in a few bugs (see details in text and S1 Table). *Indicates values are significantly different (p < 0.001) relative to other localities.

Each of the locations has a different blood source profile (Fig 4). All three localities had human, domestic animal and synanthropic animal blood sources, but opossum, an important blood source because of its relation to the sylvatic transmission cycle, was only detected in Olopa, in both, intradomestic and peridomestic bugs (S1 Fig). For Texistepeque, bird, human and mouse were found to be the most frequent blood sources detected (16%-59%). In contrast to Texistepeque, dog was the main blood source detected followed by human and mouse (19%-52%) in San Marcos de la Sierra. In Olopa, dog, bird, and human were the most detected blood sources (17%-45%). Statistical comparison of blood sources among locations indicated significant differences in bird, dog, mouse and opossum; specifically, dog detection in Texistepeque is significantly lower than in the other two localities, yet bird is significantly lower in San Marcos de la Sierra, and mouse in Olopa. When the blood meal sources are examined by ecotope, intradomestic bugs follow the same blood source profiles described above for each location. However, the low sample size of peridomestic bugs do not allow us to make statistical inference.

The 12S sequencing of samples that were positive for *T*. *cruzi* indicates additional blood meal sources: cow (in Olopa and San Marcos de la Sierra) and frog (San Marcos de la Sierra); however, the overall frequency of these new taxa was low [3 of 52 (5.7%)]. Because frogs were never seen inside houses or in the peridomicile we take this as evidence of sylvatic blood meal sources at the three locations. For Texistepeque, no new blood sources were detected, but we did find one opossum had gone undetected with opossum PCR. These results are detailed in the supplemental material (S1 Table).

### Infection with *T*. *cruzi* prevalence and its association with different ecological and life history factors at each locality

Bug infection prevalence with *T*. *cruzi* is similar for Texistepeque [30 of 71 bugs (42%)] and San Marcos de la Sierra [45 of 84 (54%)], but significantly lower in Olopa [43 of 568 (8%)] (*χ*^2^ = 136.3; *p* < 0.001). In addition, 6–25% of infected bugs either did not have a recent blood meal or had recently fed on taxa we did not assay (25% Texistepeque, 6% Olopa, and 16% San Marcos de la Sierra) (Fig 3, diagonal stripe area).

The factors associated with *T*. *cruzi* infection vary among locations (S2 Table). The three localities show significant differences with respect to only one of the four factors examined. Within each locality, the likelihood for a bug to be infected was not associated with having at least one blood source detected (S2 Table).

Ecotope only has an effect in Texistepeque where peridomestic bugs are more likely to be infected than bugs collected inside houses. For Olopa and San Marcos de la Sierra, there is no difference in the likelihood of *T*. *cruzi* infection for bugs collected from the peridomicile compared to the intradomicile (Table 3).

**Table 3:** Odds ratios for the ecological and life history factors that showed statistically significant association with bug infection with *T*. *cruzi* at each locality.

Department, municipality	Ecological and life history factor	Odds Ratio	95% CI	P-value
Santa Ana, Texistepeque	Ecotope (intradomicile/peridomicile)	0.11	0.021–0.569	< 0.01
Chiquimula, Olopa	Stage and sex (Nymphs /female)	0.335	0.146–0.769	< 0.01
	Stage and sex (Nymphs /male)	0.205	0.096–0.438	< 0.01
Intibucá, San Marcos de la Sierra	Blood meal sources detected (Human /Domesticated animals)	0.019	0.002–0.173	< 0.01
	Blood meal sources detected (Human /Synanthropic animals)	0.013	0.0006–0.255	< 0.01

The sex/stage factor had a significant effect on bugs being infected, but only in Olopa, with adults being more likely to be infected than nymphs; on the contrary there are no differences between males and females (female vs male (OD = 0.6137, 95% CI = 0.2836–1.3279, p = 0.2151). In Texistepeque and San Marcos de la Sierra, there is no difference in the likelihood of *T*. *cruzi* infection among nymphs, males and females (Table 3).

Only in the case of San Marcos de la Sierra was *T*. *cruzi* infection associated with the recent blood meal source. Bugs with evidence of human blood meals were significantly less likely to be infected with *T*. *cruzi* than bugs that fed on domestic animals (dog and pig) and blood meals from synanthropic animals (rat and mouse) (Table 3).

## Discussion

This study identified basal epidemiological similarities and differences among three locations, targeted for Ecohealth data-driven intervention. Differences are revealed in epidemiological factors including entomological indices, blood source profiles and *T*. *cruzi* infection. Because these factors are likely to influence the transmission of the Chagas disease parasite *T*. *cruzi*, they should be considered in vector control along with the household characteristics recommended by [27].

### Entomological indices

Although all three locations are known to have persistent *T*. *dimidiata* infestation both entomological indices clearly show that in Olopa the proportion of houses with bugs is higher than in the other two localities (Table 1). The infestation index (presence of adults or nymphs, or both) only indicates the possibility of a colony establishing inside a house or in a peridomestic area, or bugs migrating from the sylvatic to the intradomestic or peridomestic environment; however, the values of the colonization index (presence of nymphs with adults, or just nymphs) represents the number of houses with reproducing bugs per location.

It has been shown that risk factors for triatomine infestation differ among regions [43]. Infested houses with *T*. *dimidiata* at the three locations (Table 1) and previous studies in Jutiapa, Guatemala show most bugs were found in the intradomestic ecotope and intradomestic infestation is largely associated with poor wall conditions (no plaster or deteriorated, cracked plaster) [27, 30]. Even though house construction material (adobe, bajareque), house wall condition (no plaster or deteriorated, cracked plaster), signs of infestation (e.g., eggs, feces) and other factors (See also Table 1 from [27]) are predictors of infestation, for these three locations, this same study [27] showed that adding a random location effect improved model fit, thus confirming the variation among localities.

In contrast, peridomestic infestation tend to be associated with chicken habitats (coops or nests) [26, 44] piles of firewood [44], and coffee trees [26] in close proximity to a house, as well as tiled roofing [44]. As shown in Table 2 and [27], the most frequent peridomicile risk factor at the three locations are: chicken coops, firewood piles and construction material accumulation. Chicken coops were present in less than 45% of the houses with chickens at Texistepeque and San Marcos de la Sierra. However, more than 70% of the chicken coops were away from the house at the three localities. In addition, the frequency of having both, firewood piles and construction material accumulation, inside-adjacent to the house, were more frequent at the three localities (more than 57% and 67% respectively).

In the context of Implementation Science for Ecohealth data-driven interventions, the infestation and colonization information reported here suggest that for infested houses, spraying followed by house improvements such as wall plastering and cement floors should be performed as has been shown by [45]. This is especially a concern for Olopa, the locality with the highest infestation and colonization indices (Table 1).

However, in addition to information from the entomological indices and house risk factors, our data on blood meal source profiles and *T*. *cruzi* infection of the insect vectors also show differences among regions and provide information to guide and prioritize interventions as described below.

### Blood meal source profiles

We found relatively high feeding on humans at all three locations, highlighting the potential for *T*. *cruzi* transmission in all locations. Although there was no significant difference of human blood meals at the three locations, further study is needed to determine if the seroprevalence of *T*. *cruzi* in people is correlated to human blood meals and *T*. *cruzi* detection in vectors. This is especially a concern for Texistepeque were recently a high incidence of acute cases has been reported for Santa Ana, El Salvador [32, 46].

Host accessibility shapes a vector’s blood source profile [47] and we found that the blood meal source profiles in *T*. *dimidiata* at the three locations show statistically significant differences in bird, dog, mouse and opossum (Fig 4). Although only mammals can transmit the parasite, birds are frequent and adequate blood sources for the insect vector. The statistical analysis shows bugs feeding more frequently on birds at Texistepeque and Olopa (above 30%) than at San Marcos de la Sierra (12%). In Olopa and San Marcos de la Sierra, few bugs feeding on chickens are infected with *T*. *cruzi*, thus they appear to play a role in maintaining bug population numbers but not the parasite; whereas in Texistepeque, that many bugs that had recently fed on birds and were also positive for *T*. *cruzi*, indicates that those bugs had previously fed from an infected mammal (human or non-human) (Fig 4).

With respect to the Ecohealth data-driven interventions, the frequency of houses with birds (including chickens) with no chicken coops (82% in Texistepeque, Table 2), suggests that more attention should be focused on bird location, in particular moving the chickens into coops away from houses. With this in mind, it would be interesting to examine the effect of both chicken coop construction material and chicken coop location with respect to the house on reducing other mammal blood meals and thus *T*. *cruzi* infection as has been shown by [20] for *T*. *dimidiata* in Guatemala.

Although overall there are similar number of dogs per house in the three locations (Table 2), dogs appear to play a more important role in Olopa and San Marcos de la Sierra. For both locations, the prevalence of dog as a blood source (40%) was significantly above that in Texistepeque (5%). In studies from numerous locations across several countries, dogs are reported as the most important reservoirs of *T*. *cruzi* and are a host that coexists with people (Argentina [48], Venezuela [49] and the USA [50]). Recent studies [26] show that the number of dogs (in particular >2 per house) is an important risk factor for house infestation with *T*. *dimidiata*, and for others vectors (*Triatoma infestans*) dogs are preferred as a blood meal source over chickens [51]. In addition to being a domestic reservoir, it has been shown that dogs can become infected when roaming into sylvatic environments [52], suggesting that the prevalence of the parasite in dogs could result from vectors in both domestic and sylvatic ecotopes. In fact, the wooded areas in San Marcos de la Sierra and Olopa are more preserved than in Texistepeque (Monroy in field, personal observation). Even though overall there is on average at least one dog per house at each location (Table 2), among the houses with dogs, there is on average 2 dogs per house (SD = 1.07). Since our data show dogs are more important in the transmission cycle in San Marcos de la Sierra, for this location we suggest prioritizing controlling dog reproduction (e.g. spay or neuter) to reduce dog populations as well as the major blood source for the vector because the population abundance of a *T*. *cruzi* reservoir would be reduced.

Interestingly, opossum was only detected as a recent blood meal in Olopa (7%) (Fig 4). Opossum is considered an “ancient” host of the parasite, because it can be a reservoir and host at the same time, opossum is one of the most important sylvatic reservoirs in Chagas transmission [53]. The importance of opossum as a blood meal source for *T*. *dimidiata* was also reported for Costa Rica by [22], where rat and opossum blood meal sources were common in *T*. *cruzi* positive bugs. In Olopa, no information is available related to the abundance of sylvatic reservoirs, however, no signs of opossum were evident during our examination inside houses, this and our findings support that opossum might be moving from sylvatic to domestic ecotopes, highlighting its role as a link between the sylvatic and domestic cycle of Chagas disease.

Although mouse was significantly higher in Texistepeque and San Marcos de la Sierra (> 16%) compared to Olopa (1%), because the frequency of mouse blood meal detection is smaller than bird and dog, targeting this blood meal source would be lower priority.

Although the percent of vectors with recent blood sources was significantly different among locations, overall there was a surprisingly low percent of recent blood sources, especially in Texistepeque and San Marcos de la Sierra (T. 27%, S 38% and O 70%). These values are higher than reported in a recent study for a nearby location in El Tule, Jutiapa, Guatemala with ~15% of vectors having no recent blood source before interventions and 40% after [20]. The 12S sequencing assay indicates bugs are feeding on additional blood meal sources at all three locations: frog for San Marcos de la Sierra, cow for Olopa, and opossum for Texistepeque (S1 Table). As mentioned before, the lack of blood source detection by PCR can indicate either a recent blood meal from taxa not included in the survey or no recent blood meal [4, 20, 41]. Strong support for no recent blood meal is provided by recent studies based on mass spectrometry [54, 55] including domestic vectors from El Salvador that show DNA based methods have a short window for blood meal detection [54], it would be interesting to examine *T*. *dimidiata* where no blood sources were detected by PCR from these three localities to strengthen the Ecohealth strategies proposed by this study.

### Correlates with *T*. *cruzi* infection

Establishing if *T*. *cruzi* infection in the vector is associated with: I) the ecotope where the bug was collected, II) the ecological association of the blood source with human, or III) the stage/sex of the bug, also provides information for in shaping Ecohealth data-driven interventions. This study shows that the different factors associated with *T*. *cruzi* infection support different recommendations for the three locations.

For Texistepeque, peridomestic bugs are significantly more likely to be infected than intradomestic bugs (S2 Table). All 12 peridomestic bugs tested were collected from piles of construction material or an accumulation of firewood in the peridomicile. This reinforces other studies showing an association between infestation and accumulation of firewood in the peridomicile [57]. Since firewood piles were present in 57% of the houses surveyed in Texistepeque, Ecohealth interventions ensuring firewood piles are located away from the house should be prioritized for this location.

In contrast, for San Marcos de la Sierra, we found bugs that had fed on domestic and synanthropic animals were more likely to be infected than bugs that had fed on humans. Because 52% of the bugs had fed on dogs, Ecohealth interventions targeting dogs should be prioritized.

For Olopa, we found adult insect vectors were more likely to be infected compared to nymphs (S2 Table). This fact has been well supported elsewhere [56, 57] and this is because adults have had more time to become infected and their mobility, adult bugs can fly and are more mobile and thus can feed from a variety of blood sources, whereas nymphs likely move less and perhaps have only fed on blood sources available at the site of collection. For Olopa that the dispersing adult stage is more likely to be infected highlights the importance of Ecohealth interventions to make human dwellings less attractive to migrating bugs.

For Texistepeque and San Marcos de la Sierra the likelihood of *T*. *cruzi* infection in adult insect vectors was not significantly higher either because of small sample sizes or adults moving less at these locations. If increased sampling indicated adults are more likely to be infected than nymphs at Texistepeque and San Marcos de la Sierra (S2 Table) house improvements (e.g. wall plastering and dirt floor replacement by concrete) as suggested by [27] would be recommended for all three locations (see also [27]). Because firewood piles and construction materials have shown to be associated with triatomine infestation and at the same time were frequently located next to the house at all three localities (Table 2), we suggest that in addition to house improvements, peridomestic reorganization should be prioritized.

## Conclusions

To our knowledge, this is the first study that aims to use local information to implement an Ecohealth data-driven intervention to reduce vector transmission of *T*. *cruzi*. Our results related the presence of bugs (described by entomological indices) at both intradomestic and peridomestic ecotopes across locations and suggest that intervention is needed to decrease vector-human contact. Blood meal source analysis has shown to be a good measure of the impact of Ecohealth interventions for Jutiapa, Guatemala by [20] and similar analysis was applied to the Central American locations surveyed here with the same goal.

In this study, variation among locations in the blood source profiles and correlates with *T*. *cruzi* prevalence highlight the regional importance of domestic and synanthropic animals in disease transmission and a link between sylvatic and domestic transmission cycles. Dog is the most frequent blood source for San Marcos de la Sierra and Olopa, while humans and birds (which are blood sources but cannot become infected) in Texistepeque. In San Marcos de la Sierra, controlling dog reproduction through neutering and spaying is suggested to reduce dog populations and thus reduce the risk of the house infestation, but most important because 52% of bugs that fed on dog were *T*. *cruzi* positive then it would reduce disease incidence. It is important to remember that blood source profiles are not generalizable and represent only a snapshot in time. However, blood source profiles do provide important information to make locally relevant recommendations for Ecohealth interventions.

Our results combined with those from previous studies on blood source profiles can be used by policy makers to consider a wider breadth of vector control measures and target limited resources to locally-identified, high-impact intervention.

## Supporting information

S1 Table Three countries data file. (XLSX)

S2 Table Chi square analysis. (PDF)

S1 Fig Percent of blood meals detected in *T. dimidiata* for each of the seven taxa assayed by PCR from the three locations by ecotope. Note percentages sum to slightly more than 100 because of multiple blood meals detected in a few bugs (e.g., Texistepeque had 5 peridomestic bugs, one with both bird and rat blood meal).
(TIF)

S1 Text Opossum primer development and optimization. (DOCX)
