# Supplementary material for: Implementation science: Epidemiology and feeding profiles of the Chagas vector Triatoma dimidiata prior to Ecohealth intervention for three locations in Central America
Source: PLoS Negl Trop Dis. 2018 Nov 28;12(11):e0006952. doi: 10.1371/journal.pntd.0006952 (PMC6287883; doi:10.1371/journal.pntd.0006952)
Supplement: S2 Table — (PDF) [file pntd.0006952.s002.pdf]

Source	Risk factor			Sample size	Fisher Exact test P-value	Chi-square DF	Chi-square P-value
Texistepeque	Ecotope						
		Intradomicile	and *T. cruzi* positive	20	**0.0027**	NA	NA
			and *T. cruzi* negative	39			
		Peridomicile	and *T. cruzi* positive	10			
			and *T. cruzi* negative	2			
	Recent blood source detected*						
		Presence	and *T. cruzi* positive	12	**0.0558**	1	**0.0311**
			and *T. cruzi* negative	7			
		Absence	and *T. cruzi* positive	18			
			and *T. cruzi* negative	34			
	Stage and sex						
		Nymph	and *T. cruzi* positive	8	0.8665	2	0.8387
			and *T. cruzi* negative	12			
		Female	and *T. cruzi* positive	12			
			and *T. cruzi* negative	18			
		Male	and *T. cruzi* positive	10			
			and *T. cruzi* negative	11			
	Blood meal sources detected						
		Human	and *T. cruzi* positive	4	0.1021	NA	NA
			and *T. cruzi* negative	1			
		Domesticated	and *T. cruzi* positive	5			
			and *T. cruzi* negative	5			
		Anthropogenic	and *T. cruzi* positive	3			
			and *T. cruzi* negative	1			
		No blood meal detected	and *T. cruzi* positive	18			
			and *T. cruzi* negative	34			
Olopa	Ecotope						
		Intradomicile	and *T. cruzi* positive	37	0.2502	1	0.1956
			and *T. cruzi* negative	482			
		Peridomicile	and *T. cruzi* positive	6			
			and *T. cruzi* negative	43			
	Recent blood source detected*						
		Presence	and *T. cruzi* positive	11	0.6052	1	0.5172
			and *T. cruzi* negative	159			
		Absence	and *T. cruzi* positive	32			
			and *T. cruzi* negative	366			
	Stage and sex						
		Nymph	and *T. cruzi* positive	12	**<0.0001**	2	**<0.0001**
			and *T. cruzi* negative	316			
		Female	and *T. cruzi* positive	12			
			and *T. cruzi* negative	106			
		Male	and *T. cruzi* positive	19			
			and *T. cruzi* negative	103			
	Blood meal sources detected						
		Human	and *T. cruzi* positive	2	0.9543	NA	NA
			and *T. cruzi* negative	27			
		Domesticated	and *T. cruzi* positive	9			
			and *T. cruzi* negative	128			
		Anthropogenic	and *T. cruzi* positive	0			
			and *T. cruzi* negative	4			
		Sylvatic	and *T. cruzi* positive	1			
			and *T. cruzi* negative	11			
		No blood meal detected	and *T. cruzi* positive	32			
			and *T. cruzi* negative	366			
San Marcos de la Sierra	Ecotope						
		Intradomicile	and T. cruzi positive	38	0.7648	1	0.7209
			and T. cruzi negative	34			
		Peridomicile	and T. cruzi positive	7			
			and T. cruzi negative	5			
	Recent blood source detected*						
		Presence	and *T. cruzi* positive	29	0.6567	1	0.6066
			and *T. cruzi* negative	23			
		Absence	and *T. cruzi* positive	16			
			and *T. cruzi* negative	16			
	Stage and sex						
		Nymph	and *T. cruzi* positive	35	0.3275	2	0.3022
			and *T. cruzi* negative	25			
		Female	and *T. cruzi* positive	5			
			and *T. cruzi* negative	9			
		Male	and *T. cruzi* positive	5			
			and *T. cruzi* negative	5			
	Blood meal sources detected						
		Human	and *T. cruzi* positive	1	**<0.0001**	NA	NA
			and *T. cruzi* negative	15			
		Domesticated	and *T. cruzi* positive	27			
			and *T. cruzi* negative	11			
		Anthropogenic	and *T. cruzi* positive	5			
			and *T. cruzi* negative	1			
		No blood meal detected	and *T. cruzi* positive	16			
			and *T. cruzi* negative	16			

NA: not analyzed due to sample size of <5 .
