# Supplementary figures and images for: Implementation science: Epidemiology and feeding profiles of the Chagas vector Triatoma dimidiata prior to Ecohealth intervention for three locations in Central America
Source: PLoS Negl Trop Dis. 2018 Nov 28;12(11):e0006952. doi: 10.1371/journal.pntd.0006952 (PMC6287883; doi:10.1371/journal.pntd.0006952)

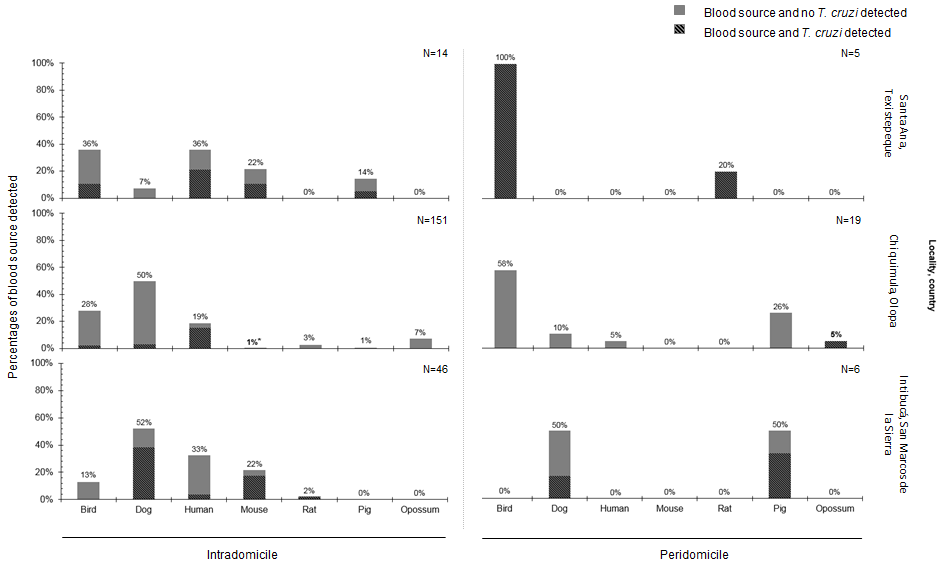

Supplement: S1 Fig — Note percentages sum to slightly more than 100 because of multiple blood meals detected in a few bugs (e.g., Texistepeque had 5 peridomestic bugs, one with both bird and rat blood meal). (TIF) [file pntd.0006952.s003.tif]
