# Supplementary material for: Implementation science: Epidemiology and feeding profiles of the Chagas vector Triatoma dimidiata prior to Ecohealth intervention for three locations in Central America
Source: PLoS Negl Trop Dis. 2018 Nov 28;12(11):e0006952. doi: 10.1371/journal.pntd.0006952 (PMC6287883; doi:10.1371/journal.pntd.0006952)
Supplement: S1 Text — (DOCX) [file pntd.0006952.s004.docx]

**Opossum primer development and optimization**

Primer design

Because *Didelphis virginiana* is the only *Didelphis* sp. within the geographic range encompassed by the study, we designed a primer specific to this species targeting the OGT gene [O-linked N-acetylglucosamine {O-GlcNAc}] using the software Primer-BLAST (Yei et al. 2012). The primer was selected based on the following parameters: Guanine/Cytosine content, Melting temperature, primer size, PCR product size, Max self-complementarity and 3’ side overlaps. In silico BLAST testing verified specificity to *D. virginiana*.

PCR standardization

PCR standardization involved primer specificity and sensitivity evaluation. Specificity was assessed by testing cross reaction with DNA extracted from chicken, rat, mouse and pig tissue, and from DNA extracted from human and dog blood. After confirming that there were no cross reactions with the species mentioned above, we tested for sensitivity using the serial dilutions: 10^-1^, 10^-2^, 10^-3^, 10^-4^, 10^-5^ and 10^-6^.

Based on the sensitivity and specificity assays, PCR conditions were denaturation at 94ºC for 2 min followed by 30 cycles at 95ºC (30 sec.), 50ºC (30 sec) and 70ºC (45 sec); followed by a final extension at 72ºC for 5 minutes. Under these conditions, opossum can be detected at concentrations as low as 10^-4^ng/uL

**Reference**

Ye J, Coulouris G, Zaretskaya I, Cutcutache I, Rozen S, Madden T (2012). Primer-BLAST: A tool to design target-specific primers for polymerase chain reaction. BMC Bioinformatics. 13:134.
